# Detection of Diazinon Residue in Honey and Honey Bee (*Apis mellifera*) in Bandar-Abbas and Meshkinshahr, Iran

**Published:** 2019-06-24

**Authors:** Fatemeh Bagheri, Hassan Vatandoost, Mansoureh Shayeghi, Yavar Rassi, Ahmad Ali Hanafi-Bojd, Abbas Rahimi-Foroushani, Alireza Razavi, Fatemeh Nikpour

**Affiliations:** 1 Department of Medical Entomology and Vector Control, School of Public Health, Tehran University of Medical Sciences, Tehran, Iran; 2 Department of Environmental Chemical Pollutants and Pesticides, Institute of Environmental Research, Tehran University of Medical Sciences, Tehran, Iran; 3 Department of Epidemiology and Biostatistics, School of Public Health, Tehran University of Medical Sciences, Tehran, Iran; 4 Department of Pathobiology, School of Public Health, Tehran University of Medical Sciences, Tehran, Iran

**Keywords:** Honey, *Apis mellifera*, Diazinon reside, HP TLC

## Abstract

**Background:**

The excessive use of pesticides for crops by farmers, their destructive effects on beneficial organisms, such as bees, have become a big problem today. This study was designed to find out if the honey bee (*Apis mellifera*) and the honey be affected by diazinon.

**Methods:**

Six hives were purchased, 3 hives in Bandar-Abbas and remaining were considered for Meshkinshahr. Plants around the hive were sprayed with diazinon at a concentration of 2/1000. Sampling took place 15d after spraying, and diazinon residue was measured by the HP TLC. The study was conducted in 2017–2018.

**Results:**

The amount of diazinon residue in honey and honey bee was measured and compared with existing studies. The amount of diazinon residue in honey bee was found to be 0.017mg/kg in Bandar Abbas, and 0.005mg/kg in Meshkinshahr hives. There was nothing in honey.

**Conclusion:**

Honey is safe for consumers.

## Introduction

Honey is a sweet, sticky and thick liquid produced by bees from nectarine flowers and contains a significant amount of minerals, vitamins, and enzymes (1). Honey is composed of main fructose (about 38.5%) and glucose (about 31.0%) (2). It is known as a nutrient and much healthier than sugar (3, 4). Honey is widely used for nutritional and medicinal purposes and can be used to treat wound infections and cancers (5). It can be used to treat cough and sore throat, stomach ulcer, earache, measles, and eye diseases (6). Honey is widely used worldwide as a food or medicine. Feeding babies with honey help to improve the memory and growth of children, as well as reduce anxiety and increase the growth performance during the life of individuals (7). Honey is used in cosmetics as well as a natural sweetener in food production. While nutritional value and different aspects of the quality of honey are important, ensuring its chemical safety is also important for consumer acceptance. Benefits of health and nutrition Honey if it is contaminated with toxic chemicals, such as contamination of honey with residues of pesticides or other environmental pollutants (8).

It can be severely exposed to various types of contaminations in the environment. Even small amounts of contaminants in the environment, such as residues of pesticides, can enter the honeybee and enter honey during processing. Since pesticides are widely used in agricultural fields and gardens to control pests and plant diseases, and hives are often found alongside the same fields and gardens, the potential for contamination of bees and consequently honey is very high. While the use of synthetic pyrethroids and carbamates as insecticides, herbicides, and fungicides are increasing, but the use of organochlorines (mainly as insecticides in food products) has been severely reduced since their ban in 1999 (9).

However, organochlorine pesticide residues are still recognized in the environment and in various types of foods because of their resistance to staying in the environment and illegal use. Even the newer generation of pesticides (artificial pyrethroids, organophosphates, and carbamates) is not as stable as the first generation organochloride, but they have very high acute toxicity. Organophosphate pesticides have been found in some areas even in fruits and vegetables that enter the market (10). All pesticides are poisonous and many of them have high potential for carcinogenesis and may even cause severe chromosomal aberrations (11). Pesticides can also interfere with endocrine disruptions, reproductive organs, fertility and the nervous system (12–15).

This study was designed to find out if the honeybee and honey are affected by diazinon.

## Materials and Methods

This study was conducted in two cities of Bandar Abbas and Meshkinshahr in 2017–2018. Meshkinshahr is located in the province of Ardebil in the northwest of Iran. This city is near the mountain and with relatively cold weather. Both plants that grow naturally in this area, as well as different herbaceous plants, will make beekeepers in the area more prevalent.

Bandar Abbas is located in Hormozgan Province in southern Iran. The climate of this city is warm and humid. The cold winters of the northern regions of Iran forced beekeepers to bring their hives to a warm tropical climate, which in winter is pleasant spring weather like Bandar Abbas.

### Measurement of residues of diazinon in honey

For this purpose, 6 hives were purchased. Plants around the hive were sprayed up to radius of 200 meters in a hive with diazinon at a concentration of 2/1000, the same conventional concentration that farmers used to control the pests. Fifteen days after spraying, honey was harvested. The control beehive was placed at a point where no spraying is done and then honey is picked up. Honey harvested from hives should be free of wax. To remove wax from honey, wax honey was placed inside stainless steel and sub filters in a clean container that was washed with conventional detergents and placed under the sun. After 24 h, wax and honey were easily separated. Subsequently, samples of harvested honey were transferred to the laboratory.

### Steps to prepare honey samples

Dissolve 50g of honey in a 50ml tube in 10ml of deionized water and place it in a blender for 1min, then acidify with acetonitrile with acidic (10ml) and 1g sodium acetate and 4g magnesium sulfate. Add water and shake for 1min. The samples were centrifuged for 4min at 2,000min. 6ml of high transparent liquid was added to 15ml polyethylene tubes containing 0.4g PSA and 0.6g magnesium sulfate without water and centrifuged at 4000 rounds per 2min. In this way, the solution was prepared to be placed on the chromatography device (16).

### Stages of preparation of bee body samples

The specimen from worker honey was transferred to disposable polyethylene bags. Honey bees were collected from honey from the honeycombs located in the furthest walls of the hive. These are typically older working honeybees, which are likely to have the most residual pesticide residues, depending on their age. All samples were transported to a laboratory in a cool box and stored at 20 °C in the laboratory until freezing.

Standard method for diazinon extraction was used (17). Subsequently, the extracted solution was measured by chromatography method and the amount of pesticide residual was measured and compared with MRL and ADI.

### Detection and measurement of diazinon in honey samples

To detect and determine the amount of pesticide, the first step is to apply a stain carried out using 20×20 plates covered with silica gel.

A standard insecticide sample of diazinon is required to stain. Spotted plates are placed at a depth of 1.5cm inside the chromatography tank. To see the spots of the plate they were placed in a UV cabinet and observed at appropriate wavelengths. Regarding standard spots and unknown spots, they can be compared with each other and determine the presence or absence of pesticides in this way qualitatively.

### HP TLC Scanner

Spot scan is done by the HP TLC. This device is designed to monitor the donometry of chromatographic and electrophoretic purposes.

After inserting the plates into the device chamber and adjusting the beam on the first spot, enter the necessary information to start the scan, such as the size of the gap, the speed of the scan, the wavelength, and the type of lamp in the corresponding section if the wavelength and the size of the correct gap are selected.

### Record spectrum

The spectrum from the detected peaks is automatically used to determine the identification of the courier or the purity comparison spectrum.

### Quantitative evaluation of results

All plates are checked after chromatography and the proper wavelength detector determines this device, and the time required for this operation is very small. Using this device, the quantitative evaluation of the material at a higher speed and more precision is performed.

## Results

The maximum residue of diazinon was in hive No. 2 in Bandar Abbas and the minimum s in Meshkinshahr No. 2 (Table 1).

**Table 1.** The number of diazinon residues in the honeybees’ body in the examined hives

**Bandar-Abbas hives**	**Diazinon residue**
Sample 1 (control)	0
Sample 2	0.025mg/kg
Sample 3	0.009mg/kg
**Meshkinshahr hives**	**Diazinon residue**
Sample 1 (control)	0
Sample 2	0.003mg/kg
Sample 3	0.007mg/kg

After analyzing the samples, the residues of diazinon in honey were not observed, and its value was considered zero. So the honey was completely clean and free of insecticide.

## Discussion

Diazinon residue in Bandar Abbas hive was 0.025 and 0.009mg/kg respectively in samples. The figures in Meshkinshar was 0.003 and 0.007mg/kg. In a study (16), 46 organochlorine, organophosphate, pyrethroid, and organonitrogen pesticides residue were analyzed in honey samples from 18 hives in 9 different locations, followed the gas chromatography. The remaining pesticides tested in 55.6% of the samples collected, and most of them were detected more and more pesticides identified belong to organochlorine and organophosphorus groups. The highest amount of pesticide residues belonged to diclofen used in the hive, because of fighting the Varroa mites, and in 38.9% of honey samples and 81.8% of identified pesticides exceeded the EU-approved honey limit, that is, MRL so the residue of diazinon was set above the limit (16), while in our study, the amount of residual diazinon was less that MRL and honey was free of pesticide (16).

In Ghana, the amount of residue of a large number of pesticides in honey was measured. Pesticides such as deltamethrin, permethrin, fenvalerate, chlorpyrophos, syphlotrin, diazinon and several other pesticides were studied. The residual of all measured pesticides is very low and below the maximum limit set by the European Union. So the analysis of residues of pesticides in honey samples shows that there are no health hazards for consumers (10). The results of this study are quite similar to the present study. In our study samples, the amount of residual diazinon was much less than detectable

The pollen of the environment can be identified with respect to the residual types of pesticides and heavy metals in honey and honey bee bodies. As one of the goals of our study was to measure the residue of the diazinon pesticide in honey and the honey bee, it is important to ensure the integrity of this essential food product and the impact of spraying the environment on the bee, which is a useful and strategic insect be aware. In our study, although some contamination was detected in the bee body, there was no contamination in honey (18).

Six villages were considered as research sites and by sampling bees and feces they measured the remaining heavy metals by using the method of Atomic absorption spectrometry and showed that the bees changed to chemical in the environment in which they live, especially the increase in the number of heavy metals in soil, air and plants, and the residues of these substances in the body of the bee can represent the presence of these substances in nature. Although there may not be honey in the bee product, so these studies indicate that the bees are a reliable indicator of the cleanliness and lack of environment of any chemical (19).

The l residue of different pesticides was investigated in local honey samples produced from different places in Jordan and from imported honey during 1995–1995. The remaining 50 pesticides were determined in 26 samples of honey. The highest amount of pesticides in the samples was assigned to the chlorine group. Lower levels remained in some organophosphates and pyrothyroids. In the 11 samples examined, the remaining αHCH, βHCH, and lindane residues were detectable. In some of the samples, DDT, heptachlor, heptachlor oxide, dieldrin, and aldrin were also detected. The fluvalinate residue was found only in 4 honey samples. The amitraz residues, tetradifon and bromopropylate were not detectable in honey samples. The measurement of these pesticides in honey was carried out using the GC method. The similarity of this study with our finding is that the diazinon pesticides in honey samples were zero (20).

## Conclusion

There was no pesticide residue in honey so it seems honey is safe for consumers.
